# Supplementary material for: Cost analysis of a nationwide typhoid conjugate vaccine campaign in Burkina Faso
Source: PLoS One. 2026 Jun 10;21(6):e0351148. doi: 10.1371/journal.pone.0351148 (PMC13252793; doi:10.1371/journal.pone.0351148)
Supplement: S1 File — (DOCX) [file pone.0351148.s001.docx]

**Supporting Information**

**Appendix 1. Secondary data**

Table 1 and Table 2 present the replacement price, assumed useful life years of capital items and salary scales for campaign personnel.

**Table 1. Replacement prices and useful life years for vehicules and equipment**

| **Replacement prices and useful life years (ULY) for equipment and vehicle** | |
| --- | --- |
| Mobile phone | 11,000 F CFA ULY 2 years |
| Smartphone | 102,000 F CFA ULY 2 years |
| Desktop | 375,000 F CFA ULY 5 years |
| Laptop | 345,000 F CFA ULY 5 years |
| Tablet | 140,000 F CFA ULY 2 years |
| Megaphone | 44,000 F CFA ULY 5 years |
| Printer | 67,000 F CFA ULY 5 years |
| Incinerator (health facility) | 1,000,000 F CFA ULY 10 years |
| Ambulance | 47,500,000 F CFA ULY 6 years |
| Refrigerated truck | 55,900,000 F CFA ULY 10 years |
| Car | 32,500,000 F CFA ULY 6 years |
| Motorcycle | 656,250 F CFA ULY 4 years |
| Motorcycle (Tricycle) | 925,000 F CFA ULY 4 Years |
| Bike | 52,000 F CFA ULY 4 years |
| Generator | 1,000,000 F CFA ULY 5 years |
| **Replacement prices and useful life years (ULY) for cold chain equipment** | |
| Cold box | $175 ULY 5 years |
| Ice pack | $3.50 ULY 2 years |
| Vaccine carrier | $1.8 - $34.9 depending on size ULY 5 years |
| Refrigerators and cold rooms | As per model and UNICEF pricelist ULY 10 years |

**Table 2 Salary scales for campaign personnel**

| **Personnel (grade)** | **Monthly Salary** |
| --- | --- |
| **Staff not concerned by the hospital public service reform** | |
| General practitioner (PC) | 123,932 F CFA |
| Specialist (PA) | 184,149 F CFA |
| Pharmacist (P) | 184,149 F CFA |
| Specialized nurse (A) | 123,932 F CFA |
| Midwife (B) | 100,233 F CFA |
| Nurse (B) | 100,233 F CFA |
| "Agent de santé communautaire" (C) | 79,837 F CFA |
| Itinerant health agent or "Agent itinérant de santé" (C) | 79,837 F CFA |
| Midwife assistant or "Accoucheuse" (D) | 63,131 F CFA |
| **Personnel (grade)** | **Monthly Salary** |
| **Staff concerned by the hospital public service reform** | |
| General practitioner (U2) | 170,163 F CFA |
| Specialist (U1) | 221,057 F CFA |
| Pharmacist (U2) | 221,057 F CFA |
| Specialized nurse (N) | 170,163 F CFA |
| Midwife (M) | 120,241 F CFA |
| Nurse (M) | 120,241 F CFA |
| "Agent de santé communautaire" (S) | 95,765 F CFA |
| Itinerant health agent or "Agent itinérant de sante" (S) | 95,765 F CFA |
| Midwife assistant or "Accoucheuse" (F) | 75,563 F CFA |
| Volunteer | 45,000 F CFA |

**Appendix 2. Overview of the campaign costs estimation**

- **Allocations rules**

Table 1 presents all activities planned for the campaign and specifies the financial and economic cost categories associated with each activity included in the study. These activities align with those outlined in existing guidelines for estimating vaccination costs (Clarke-Deelder et al., 2019). The table also indicates the program levels at which each activity applies (column 4).

Data on quantities of resources used for each activity were collected through Interviews conducted at multiple administrative levels, including health facilities, districts, regions, and the national level. For shared resources, allocation (tracing) factors were collected during the costing interviews to appropriately attribute costs to TCV delivery. These tracing factors included the proportion of staff time dedicated to the campaign, the share of vehicle and equipment capacity utilized during the campaign, and the number of doses administered or the volume of other vaccines delivered during the campaign.

**Table 1. Costs of the TCV vaccination campaign by activity and detailed overview of financial and economic cost estimation components.**

| **Activity** | **Financial cost categories/items** | **Economic cost categories/items** | **Level (HF, District, Region, National)** | **Detailed description of cost components** |
| --- | --- | --- | --- | --- |
| Planning and programme/campaign management | Per diem and travel allowances; fuel costs and vehicle maintenance; venue rental, catering and other costs; communication costs | Value of staff time devoted to planning and programme/campaign management; per diem and travel allowances; fuel and maintenance; vehicle depreciation; venue rental, catering and other costs | All levels | Number of staff involved (n); frequency of the activity (f); time spent per occurrence (t); cost of staff (salary and benefits) per unit of time (c)  *Staff time = n × f × t × c;*  Number and duration of meetings; number of participants receiving per diem; rates by type of staff; vehicle types and replacement cost; Utilization rate of resources for TCV campaign activities  Fuel and maintenance costs; expenditures on venue rental and communication |
| Social mobilization / Communication activities | Per diem and travel allowances; printing of communication materials; distribution of communication materials;  Fuel and vehicle maintenance; Production and broadcasting of TV/radio spots; Awareness activities | Value of staff, teachers and volunteers’ time devoted to social mobilization/ communication activities;  Per diem; Printing;  Fuel and maintenance; Vehicle depreciation; Production and broadcasting of TV/radio spots | All levels | *Staff time = n × f × t × c*; number of meetings; participants receiving per diem;  Cost of printing materials;  Cost of broadcasting media messages |
| Training | Per diem and travel allowances;  Supplies;  Production and printing of training materials;  Venue rental;  Trainer costs | Value of staff time devoted to training and development of training materials;  Per diem; Supplies; Printing;  Venue rental; Trainer costs | All levels | *Staff time = n × f × t × c*; number and duration of trainings; participants receiving per diem;  Cost of supplies;  Cost of printing materials;  Venue rental costs; Catering  Trainer payments |
| Vaccine collection, distribution and storage | Per diem and travel allowances for distribution/collection;  Fuel and maintenance;  Energy costs for storage equipment | Value of staff time devoted to collection, distribution and storage;  Per diem;  Vehicle and equipment depreciation;  Ffuel and maintenance; Energy costs | All levels | *Staff time = n × f × t × c*; Number and duration of trips devoted to collection, distribution and storage ; number of staff involved;  Vehicle type and replacement cost;  Fuel and vehicle maintenance costs;  Cold chain equipment, energy consumption and maintenance; tracing factors |
| Service delivery | Per diem and allowances for vaccination teams; Supplies (e.g. cotton); Fuel and vehicle maintenance; Additional supplies paid by facility/district;  Venue rental; community health workers | Value of staff time devoted to vaccination activities;  Per diem; Supplies;  Vehicle depreciation;  Fuel and maintenance; Additional supplies | Health facility | *Staff time = n × f × t × c*; Number and duration of sessions; number of staff;  Transport modes;  Fuel and maintenance costs;  Quantities and costs of supplies;  Additional expenditures; |
| Supervision | Per diem and travel allowances;  Fuel and maintenance | Value of staff time devoted to supervision; Vehicle depreciation;  Per diem;  Fuel and maintenance | National, Region, District | *Staff time = n × f × t × c*; number and duration of supervision visits; number of staff;  Vehicle type and replacement cost;  Fuel and maintenance costs |
| Record keeping and reporting | Tally sheets; session recording forms; Monthly reporting forms; vaccination cards; registers; pens and pencils | Value of staff time devoted to record keeping, reporting, monitoring and evaluation;  Tally sheets; session recording forms; Monthly reporting forms; vaccination cards; registers | Health facility, District, Region | Staff time = n × f × t × c; cost of stationery;  cost of vaccination cards and reporting tools |
| Waste management | Fuel for incinerating vaccination waste | Value of staff time devoted to waste management; Fuel;  Incinerator depreciation; Transport costs | Health facility, District, Region | Staff time = n × f × t × c; fuel costs; incinerator replacement cost; transport and vehicle costs |
| AEFI management | Per diem and travel allowances;  Fuel and maintenance;  Vehicle depreciation  Supplies | Value of staff time devoted to AEFI management; Fuel;  Vehicle depreciation; | Health facility | Staff time = n × f × t × c; number and duration of AEFI management visits; number of staff;  Vehicle type and replacement cost;  Fuel and maintenance costs  Supplies |
| Crisis response and management | Per diem and travel allowances; fuel and maintenance; media production and broadcasting; press conferences; newspaper articles; speaker fees | Value of staff time devoted to crisis response; per diem; fuel and maintenance; media production; press conferences; publications; expert fees | All levels | *Staff time = n × f × t × c*; number of days for crisis response; participants receiving per diem; Vehicle used; communication costs (TV/radio, press, publications) |

- **Methods used to annualize capital costs and assumptions about useful life**

To account for their use over multiple years, capital items (e.g. cold chain equipment, vehicles, and durable equipment) were annualized using an annualization factor (see formula below) with a discount rate (r) and an assumed useful life (n) for capital items. This annualization factor was applied to the replacement cost of each capital item. The resulting annualized cost was allocated to the campaign based on the proportion of time the asset was used during campaign activities.

$Annualization factor=\frac{{(1+r)}^{n-1}}{(r x {(1+r)}^{n}}$

(1)

**Appendix 3. Sensitivity and scenario analysis Results**

We conducted a one-way sensitivity analysis to assess the robustness of cost estimates. Key parameters including volunteer wage and vehicle and equipment replacement costs were varied ±20%. This range was selected as a plausible variation in the absence of precise parameter uncertainty and is consistent with standard practice in health economic evaluations and immunization costing studies (Boonstoppel et al. 2021, Drummond et al. 2015 ). In addition, the discount rate was varied from 1% to 5%.

Figure 1 presents a tornado diagram illustrating the impact of parameters on the economic cost per dose. The analysis indicates that the valuation of volunteer time had the greatest influence on the estimated cost per dose. Varying this parameter by ±20% resulted in a 3% change in the economic cost per dose, ranging from $ 2.13 (95% CI: $1.62–$2.46) to $ 2.19 (95% CI: $1.67–$2.52).





**Fig. 1 One-way sensitivity analysis on key parameter assumptions**

Excluding inaccessible health facilities from the analysis did not change the financial cost per dose, which remained at $ 0.47. However, the economic cost per dose increased by 1.4%, from $ 2.16 in the base-case analysis to $ 2.19 (95% CI: $1.67–$2.53).
