# Supplementary material for: Cost analysis of a nationwide typhoid conjugate vaccine campaign in Burkina Faso
Source: PLoS One. 2026 Jun 10;21(6):e0351148. doi: 10.1371/journal.pone.0351148 (PMC13252793; doi:10.1371/journal.pone.0351148)
Supplement: S3 File — (DOCX) [file pone.0351148.s003.docx]

**S3. Supporting information for data availability.**

The datasets and variable codebook can be found here: <https://doi.org/10.7910/DVN/CHBLZK>

The Online supporting information contains the following files:

1. "Health facility level Dataset.tab"
2. "District level Dataset.tab"
3. "Region level Dataset.tab"
4. "National level Dataset.tab"
5. "Variable_Codebooks_All_Levels.xlsx "
