# Supplementary material for: Cost analysis of a nationwide typhoid conjugate vaccine campaign in Burkina Faso
Source: PLoS One. 2026 Jun 10;21(6):e0351148. doi: 10.1371/journal.pone.0351148 (PMC13252793; doi:10.1371/journal.pone.0351148)
Supplement: Attachment — Submitted filename: PLOS Article review on Vaccine Burkina Faso.odt [file pone.0351148.s004.odt]

Title: Cost analysis of a nationwide typhoid conjugate vaccine campaign in Burkina Faso
This study is very important in ensuring that scarce resources are judiciously used especially in resource limited country like Burkina Faso. 

This is my comment.
The definition of financial cost and economic cost should be included in the introduction because it will give the reader a better understanding of the cost estimate of administering the vaccine in Burkina Faso.
