# Supplementary material for: Cost analysis of a nationwide typhoid conjugate vaccine campaign in Burkina Faso
Source: PLoS One. 2026 Jun 10;21(6):e0351148. doi: 10.1371/journal.pone.0351148 (PMC13252793; doi:10.1371/journal.pone.0351148)
Supplement: Attachment — Submitted filename: Responses to reviewers.docx [file pone.0351148.s007.docx]

**Dear Editor,**

**We thank you, the PlosOne editorial team and all the reviewers for the insightful and constructive comments to our submission. We have revised the manuscript substantially in response to the comments, to enhance its clarity, transparency, and interpretability. Below, we respond to each comment point-by-point.**

**Editor and reviewers comments are reproduced in regular font, followed by our responses in bold; important substantive changes to the manuscript are also included in italic.**

Editor comments
Thank you for the study; it is quite an interesting manuscript to read. However, I have some few concerns.
First, there is a need to discuss what 'financial and economic cost' means. You need to bring that in your introduction and also state how it was determined in the method section, because you used it quite a lot in the abstract.
Also, there are inconsistencies in the denominator used, so you need to revisit that and make it consistent.

**We added the definition of financial cost and economic cost in the introduction section and explain how they were estimated in the method section and in supporting information.**

**Also, we thank the editor for identifying this inconsistency in the denominator. The correct one is 10,552,806 doses, corresponding to the validated dataset used for all cost analyses.**

**Review Comments to the Author**

**Reviewer #1: 
We thank the reviewer for the encouraging and thoughtful review, and for recognizing the major strengths, and policy relevance of the study.**

1. Clarify key microcosting assumptions
The manuscript would benefit from additional detail on:
• How shared or overhead costs (e.g., planning, supervision) were allocated across doses or administrative levels.

**Response to Reviewer #1: The purpose of the study is to estimate TCV campaign specific costs. We estimate these costs at each administrative level by quantifying ressources used for each campaign activity. For shared resources, tracing factors (allocation rules) were used to attribute costs to TCV campaign. These tracing factors included staff time allocated to the campaign, percentage of vehicle and equipment capacity utilized during the campaign, or the number of doses and the volume of other vaccines administered or interventions delivered as part of the campaign.**

• Methods used to annualize capital costs and assumptions about useful life.

**Response to Reviewer #1: To reflect their use over multiple years, capital items (e.g. cold chain equipment, vehicles, and durable equipment) were annualized using an annualization factor (see formula below) with a discount rate (r) and an assumed useful life (n) for capital items. The annualized cost was allocated to the campaign based on the proportion of time the asset was used during campaign activities.**

(1)

$Annualization factor=\frac{{(1+r)}^{n-1}}{(r x {(1+r)}^{n}}$

• How staff time and opportunity costs were valued for economic cost estimates.

**Response to the reviewer #1: For economic cost estimates, staff time was valued using an opportunity cost approach. Time allocated to campaign activities was obtained through interviews with health personnel and program managers and multiplied by the corresponding gross salary to estimate the economic value of personnel time.**

These clarifications could be addressed succinctly in a methods appendix or supplementary table and would improve reproducibility.

**Response to the reviewer #1: We added a supplementary file explaining these clarifications on the estimation of the campaign costs.**

2. Expand description of uncertainty estimation
While confidence intervals are reported, the paper should briefly explain:
• The statistical method used to generate them (e.g., bootstrapping, sampling-based variance).
• What sources of uncertainty are captured (sampling only vs. parameter uncertainty).
This would strengthen readers’ confidence in the precision of the estimates.

**We have revised the Methods section (lines 158-166) to clearly describe how confidence intervals were generated. Specifically, 95% confidence intervals were calculated using sampling-based variance derived from the study sample, with standard errors estimated from the variability in cost estimates across sampled administrative levels. Confidence intervals were constructed using the normal approximation. These intervals capture sampling uncertainty related to the selection of study sites, but do not incorporate parameter uncertainty. Therefore we conducted one-way sensitivity analyses on key parameters (discount rate, volunteer wage and vehicle and equipment replacement costs). This clarification has been added to the Methods section: «*Uncertainty around cost estimates was assessed using sampling-based variance derived from the study sample. Standard errors were calculated based on the variability in cost estimates across the sampled administrative levels. 95% confidence intervals were constructed using the normal approximation. Parameter uncertainty was explored through one-way sensitivity analysis on key assumptions, including the discount rate, valuation of volunteer time, and replacement costs of capital items (supplementary file ). Also, we conduct a scenario analysis by removing the replaced facilities due to security constraints from the analysis.*»**

3. Improve discussion of generalizability
Given the unique security and logistical context of Burkina Faso, the discussion could more explicitly address:
• Which cost components are likely context-specific.
• Which findings (e.g., dominance of HR and per diems) are likely transferable to other low-income settings.
This would help readers appropriately interpret and apply the results elsewhere.

**We appreciated the suggestion and have expanded the Discussion section to include cost components likely context-specific and those transferable to low-and-middle income countries (see lines 332-338): «*The generalizability of these findings should be interpreted in light of the specific contextual features of Burkina Faso.* *While some cost drivers—such as increased transport and logistical costs in conflict-affected areas—are context-specific to Burkina Faso, key findings are likely transferable. In particular, the dominance of human resources and per diem payments as major cost drivers, as well as variations by delivery strategy and economies of scale, are consistent with evidence from other low-income settings.*»**

4. Minor clarity and consistency issues

A careful editorial pass could improve:
• Consistency in terminology across sections (e.g., delivery strategy labels).
• Clearer signposting between financial vs. economic cost results.
• Minor typographical and formatting issues.
**All minor comments have been addressed as follows: We have improved the clarity of presentation by consistently distinguishing between financial and economic costs throughout the manuscript. Separate subheadings and explicit terminology are now used to guide the reader, and additional explanations have been included to clarify the conceptual differences between these two cost measures.**

**Reviewer #2:**

**We thank the reviewer for the constructive review of the manuscript. Below we address the clarifications suggested.**

Briefly describe the method used to derive confidence intervals:

**We have revised the Methods section (lines 158-166) to clearly describe how confidence intervals were generated. Specifically, 95% confidence intervals were calculated using sampling-based variance derived from the study sample, with standard errors estimated from the variability in cost estimates across sampled administrative levels. Confidence intervals were constructed using the normal approximation. These intervals capture sampling uncertainty related to the selection of study sites, but do not incorporate parameter uncertainty. Therefore we conducted one-way sensitivity analyses on key parameters (discount rate, volunteer wage and vehicle and equipment replacement costs). This clarification has been added to the Methods section: «*Uncertainty around cost estimates was assessed using sampling-based variance derived from the study sample. Standard errors were calculated based on the variability in cost estimates across the sampled administrative levels. 95% confidence intervals were constructed using the normal approximation. Parameter uncertainty was explored through one-way sensitivity analysis on key assumptions, including the discount rate, valuation of volunteer time, and replacement costs of capital items (supplementary file ). Also, we conduct a scenario analysis by removing the replaced facilities due to security constraints from the analysis.*»**

Clarify the potential impact of replacing inaccessible health facilities due to security constraints. **In the study limitations section (lines 368-373) we have a short paragraph acknowledging that five health facilities were inaccessible due to security constraints and were replaced with accessible facilities considered comparable in terms of cost structure or campaign implementation (similar catchment area population, number of doses delivered). Moreover, we added a scenario analysis excluding these health facilities. This analysis indicated that their replacement had no substantial impact on either financial or economic costs estimates (See lines 270-274).**

**Reviewer #3:** 
**We thank the reviewer for the positive evaluation of the manuscript’s relevance, strengths, as well as for the detailed and constructive suggestions.**

**Below we address the suggested comments.**

1. Inconsistent Dose Denominators
Issue: The manuscript reports conflicting total dose numbers:

Abstract and line 197: 10,552,806 doses
Abstract line 38: "10.5 million doses"
Table 2: 11,422,855 doses nationally

Impact: All per-dose cost calculations depend on the correct denominator. This inconsistency undermines confidence in the primary outcome.

Please Reconcile these figures and explain any discrepancy (e.g., incomplete reporting from non-sampled districts)
Use consistent denominator throughout
Recalculate all per-dose costs if necessary
Verify Table 3 calculations align with corrected denominators

**We thank the reviewer for identifying this inconsistency. The correct denominator is 10,552,806 doses, corresponding to the validated dataset used for all cost analyses. The higher figure previously reported in Table 2 (11,422,855 doses) reflected an earlier, uncleaned version of the national dataset and has now been corrected. All per-dose cost estimates, including those in the Abstract and Table 3, were calculated using the correct denominator; therefore, no recalculation was required.**

2. Uncertainty Quantification Insufficient
Issue: 95% confidence intervals are presented but methodology is not described. No sensitivity analyses are provided despite multiple assumptions (discount rate, volunteer time valuation, vehicle replacement costs, useful life).

I suggest an additional Methods subsection describing CI calculation (bootstrap? parametric assumptions? accounting for clustering?)
Conduct one-way sensitivity analyses on key parameters:

Discount rate (0%, 3%, 5%)
Volunteer wage assumptions
Vehicle/equipment replacement costs

**We have revised the Methods section to clearly describe how confidence intervals were generated. Specifically, 95% confidence intervals were calculated using sampling-based variance derived from the study sample, with standard errors estimated from the variability in cost estimates across sampled administrative levels. Confidence intervals were constructed using the normal approximation. These intervals capture sampling uncertainty related to the selection of study sites, but do not incorporate parameter uncertainty. Therefore we conducted one-way sensitivity analyses on key parameters (discount rate, volunteer wage and vehicle and equipment replacement costs). This clarification has been added to the Methods section: «*Uncertainty around cost estimates was assessed using sampling-based variance derived from the study sample. Standard errors were calculated based on the variability in cost estimates across the sampled administrative levels. 95% confidence intervals were constructed using the normal approximation. Parameter uncertainty was explored through one-way sensitivity analysis on key assumptions, including the discount rate, valuation of volunteer time, and replacement costs of capital items (supplementary file ). Also, we conduct a scenario analysis by removing the replaced facilities due to security constraints from the analysis.*»**

3. Capital Cost Treatment and Shared Infrastructure
Issue: The authors acknowledge excluding "depreciation of long-term infrastructure" (line 309-310) but do not specify which items. It's unclear whether this represents:

Incremental campaign costs only, or
Full economic costs excluding pre-existing infrastructure

Impact: Affects comparability with other studies and interpretation of economic costs.

Specify which capital items were excluded (cold chain equipment? storage facilities? EPI buildings?)
State clearly whether the perspective is incremental campaign cost vs. average cost including shared infrastructure
Justify exclusions based on whether infrastructure was campaign-specific or shared with routine immunization
If feasible, provide sensitivity estimate including shared infrastructure costs.

**We have revised the manuscript to clarify the costing perspective and the treatment of capital and shared infrastructure costs (lines 333-334). The analysis adopts an incremental campaign costing perspective, aiming to capture the additional resources required to implement the TCV campaign, rather than the full economic cost of the immunization system.**

**Accordingly, long-term infrastructure costs were excluded, specifically EPI buildings and office infrastructure. These items were excluded because they were not procured nor expanded specifically for the campaign and are shared with routine immunization activities. We have now explicitly stated this in the Methods section and clarified that the reported economic costs include annualized capital costs for campaign-specific items only (e.g., vehicles and equipment mobilized for the campaign where applicable).**

MINOR CONCERNS
Methods and Reporting

Line 72: Specify which TCV vaccine (Typbar TCV® or TYPHIBEV®) was used in Burkina Faso

**In Burkina Faso, TYPHIBEV® was used for the campaign.**

Lines 97-98: Standardize terminology as "microcosting" (no hyphen) throughout

**We used a standard terminology «microcosting» throughout the manuscript.**

Line 120: Describe replacement protocol for 5 inaccessible CSPS more rigorously. What specific matching criteria were used? Conduct sensitivity analysis excluding these sites.

**We added a sentence in the methods section to describe more rigorously the replacement protocol of inaccessible facilities as follows (lines 123-127): «*Due to security concerns, we replaced five inaccessible CSPS with alternative facilities with similar characteristics. The matching criteria were based on sampling weights derived from catchment area population size and the number of doses delivered, as well as stratification by urban versus rural location.*»**

**Furthermore we conducted a scenario analysis excluding these sites This analysis indicated that there was no substantial impact on either financial or economic costs estimates (See lines 270-274).**

Line 153: Clarify how oral consent was documented
Add to Methods: "Reporting follows CHEERS 2022 guidelines for economic evaluations" and include completed checklist as supplementary file

**Oral consent was obtained prior to each interview after the study objectives and procedures were read aloud to participants. Consent was documented by the data collector’s signature on a standardized form confirming that the information had been provided and that the participant agreed to take part in the study. Also, we added to methods section, a sentence related to CHEERS guidelines and included completed CHEERS checklist as a supplementary file.**

**Reviewer #4:**

**We thank the reviewer for the constructive review of the manuscript**

The definition of financial cost and economic cost should be included in the introduction because it will give the reader a better understanding of the cost estimate of administering the vaccine in Burkina Faso.

**We added the definition of financial cost and economic cost in the introduction section to help the reader.**
