# Supplementary material for: Cost analysis of a nationwide typhoid conjugate vaccine campaign in Burkina Faso
Source: PLoS One. 2026 Jun 10;21(6):e0351148. doi: 10.1371/journal.pone.0351148 (PMC13252793; doi:10.1371/journal.pone.0351148)
Supplement: Attachment — Submitted filename: PLOS reviewer.docx [file pone.0351148.s006.docx]

**Cost analysis of a nationwide typhoid conjugate vaccine campaign in Burkina Faso.**

The issue of definition of some terms had been addressed.

Thanks
