# Supplementary material for: Cost analysis of a nationwide typhoid conjugate vaccine campaign in Burkina Faso
Source: PLoS One. 2026 Jun 10;21(6):e0351148. doi: 10.1371/journal.pone.0351148 (PMC13252793; doi:10.1371/journal.pone.0351148)
Supplement: Attachment — Submitted filename: Responses to reviewers_V2.docx [file pone.0351148.s008.docx]

**Dear Editor,**

**We thank you, the PlosOne editorial team and all the reviewers for the constructive comments to our submission. Below, we respond to each comment.**

**Editor and reviewers comments are reproduced in regular font, followed by our responses in bold.**

 Review Comments to the Author

Reviewer #3: The authors have substantively improved the manuscript and addressed most prior concerns. Remaining issues are mostly editorial

**We thank the reviewer for their positive assessment and constructive comments. We have carefully reviewed the manuscript and addressed all remaining editorial issues, including formatting consistency, tense agreement, and citation style. Changes are highlighted in the tracked-changes version of the manuscript.**

Reviewer #4: The topic Cost analysis of a nationwide typhoid conjugate vaccine campaign in Burkina Faso.
The issues of definition of some terms were addressed and the define them in the introduction section of the paper.
I believe that the article can be published.

**We thank the reviewer for their review and positive recommendation.**
