# Supplementary material for: Cost analysis of a nationwide typhoid conjugate vaccine campaign in Burkina Faso
Source: PLoS One. 2026 Jun 10;21(6):e0351148. doi: 10.1371/journal.pone.0351148 (PMC13252793; doi:10.1371/journal.pone.0351148)
Supplement: Peer review history

# Peer review: Decision Letter 0

**DOI:** 10.1371/journal.pone.0351148.r001

**Authors:** Leonard Uzairue

4 Mar 2026

PONE-D-25-66817

Cost analysis of a nationwide typhoid conjugate vaccine campaign in Burkina Faso

PLOS One

Dear Dr. Koulidiati,

Thank you for submitting your manuscript to PLOS ONE. After careful consideration, we feel that it has merit but does not fully meet PLOS ONE’s publication criteria as it currently stands. Therefore, we invite you to submit a revised version of the manuscript that addresses the points raised during the review process.

Kindly ensure to examine all the comments from the reviewers and provide a point by point responses to their comments.

Please submit your revised manuscript by Apr 18 2026 11:59PM. If you will need more time than this to complete your revisions, please reply to this message or contact the journal office at plosone@plos.org. When you're ready to submit your revision, log on to https://www.editorialmanager.com/pone/ and select the 'Submissions Needing Revision' folder to locate your manuscript file.

Please include the following items when submitting your revised manuscript:

- A letter that responds to each point raised by the academic editor and reviewer(s). You should upload this letter as a separate file labeled 'Response to Reviewers'.
- A marked-up copy of your manuscript that highlights changes made to the original version. You should upload this as a separate file labeled 'Revised Manuscript with Track Changes'.
- An unmarked version of your revised paper without tracked changes. You should upload this as a separate file labeled 'Manuscript'.

If you would like to make changes to your financial disclosure, please include your updated statement in your cover letter. Guidelines for resubmitting your figure files are available below the reviewer comments at the end of this letter.

If applicable, we recommend that you deposit your laboratory protocols in protocols.io to enhance the reproducibility of your results. Protocols.io assigns your protocol its own identifier (DOI) so that it can be cited independently in the future. For instructions see: https://journals.plos.org/plosone/s/submission-guidelines#loc-laboratory-protocols. Additionally, PLOS ONE offers an option for publishing peer-reviewed Lab Protocol articles, which describe protocols hosted on protocols.io. Read more information on sharing protocols at https://plos.org/protocols?utm_medium=editorial-email&utm_source=authorletters&utm_campaign=protocols.

We look forward to receiving your revised manuscript.

Kind regards,

Leonard Ighodalo Uzairue, PhD

Academic Editor

PLOS One

Journal Requirements:

When submitting your revision, we need you to address these additional requirements.

1.Please ensure that your manuscript meets PLOS ONE's style requirements, including those for file naming. The PLOS ONE style templates can be found at

https://journals.plos.org/plosone/s/file?id=wjVg/PLOSOne_formatting_sample_main_body.pdf and

https://journals.plos.org/plosone/s/file?id=ba62/PLOSOne_formatting_sample_title_authors_affiliations.pdf

2. We note that the grant information you provided in the ‘Funding Information’ and ‘Financial Disclosure’ sections do not match.

When you resubmit, please ensure that you provide the correct grant numbers for the awards you received for your study in the ‘Funding Information’ section.

3. Thank you for stating the following financial disclosure:

“This work was undertaken as part of the Typhoid Vaccine Acceleration Consortium (TyVAC). TyVAC is a partnership between the Center for Vaccine Development and Global Health at the University of Maryland School of Medicine; the Oxford Vaccine Group at the University of Oxford; and PATH, an international nonprofit. This work was supported, in whole or in part, by the Gates Foundation [INV-030857]. Under the grant conditions of the Gates Foundation, a Creative Commons Attribution 4.0 Generic License has already been assigned to the Author Accepted Manuscript version that might arise from this submission.

J-LK, as an independent consultant and RLZ, DAVYCAS International staff, received consulting fees from PATH.”

Please state what role the funders took in the study.  If the funders had no role, please state: "The funders had no role in study design, data collection and analysis, decision to publish, or preparation of the manuscript."

If this statement is not correct you must amend it as needed.

Please include this amended Role of Funder statement in your cover letter; we will change the online submission form on your behalf.

4. For studies involving third-party data, we encourage authors to share any data specific to their analyses that they can legally distribute. PLOS recognizes, however, that authors may be using third-party data they do not have the rights to share. When third-party data cannot be publicly shared, authors must provide all information necessary for interested researchers to apply to gain access to the data. (https://journals.plos.org/plosone/s/data-availability#loc-acceptable-data-access-restrictions)

For any third-party data that the authors cannot legally distribute, they should include the following information in their Data Availability Statement upon submission:

1) A description of the data set and the third-party source

2) If applicable, verification of permission to use the data set

3) Confirmation of whether the authors received any special privileges in accessing the data that other researchers would not have

4) All necessary contact information others would need to apply to gain access to the data

5. PLOS requires an ORCID iD for the corresponding author in Editorial Manager on papers submitted after December 6th, 2016. Please ensure that you have an ORCID iD and that it is validated in Editorial Manager. To do this, go to ‘Update my Information’ (in the upper left-hand corner of the main menu), and click on the Fetch/Validate link next to the ORCID field. This will take you to the ORCID site and allow you to create a new iD or authenticate a pre-existing iD in Editorial Manager.

6. We note you have included a table to which you do not refer in the text of your manuscript. Please ensure that you refer to Table 3 in your text; if accepted, production will need this reference to link the reader to the Table.

7. If the reviewer comments include a recommendation to cite specific previously published works, please review and evaluate these publications to determine whether they are relevant and should be cited. There is no requirement to cite these works unless the editor has indicated otherwise.

Additional Editor Comments:

Thank you for the study; it is quite an interesting manuscript to read. However, I have some few concerns.

First, there is a need to discuss what 'financial and economic cost' means. You need to bring that in your introduction and also state how it was determined in the method section, because you used it quite a lot in the abstract.

Also, there are inconsistencies in the denominator used, so you need to revisit that and make it consistent.

[Note: HTML markup is below. Please do not edit.]

Reviewers' comments:

Reviewer's Responses to Questions

**Comments to the Author**

1. Is the manuscript technically sound, and do the data support the conclusions?

The manuscript must describe a technically sound piece of scientific research with data that supports the conclusions. Experiments must have been conducted rigorously, with appropriate controls, replication, and sample sizes. The conclusions must be drawn appropriately based on the data presented.

Reviewer #1: Yes

Reviewer #2: Yes

Reviewer #3: Yes

Reviewer #4: Yes

**********

2. Has the statistical analysis been performed appropriately and rigorously?

Reviewer #1: Yes

Reviewer #2: Yes

Reviewer #3: I Don't Know

Reviewer #4: Yes

**********

3. Have the authors made all data underlying the findings in their manuscript fully available?

The PLOS Data policy requires authors to make all data underlying the findings described in their manuscript fully available without restriction, with rare exception (please refer to the Data Availability Statement in the manuscript PDF file). The data should be provided as part of the manuscript or its supporting information, or deposited to a public repository. For example, in addition to summary statistics, the data points behind means, medians and variance measures should be available. If there are restrictions on publicly sharing data—e.g. participant privacy or use of data from a third party—those must be specified.

Reviewer #1: No

Reviewer #2: Yes

Reviewer #3: Yes

Reviewer #4: Yes

**********

4. Is the manuscript presented in an intelligible fashion and written in standard English?

PLOS ONE does not copyedit accepted manuscripts, so the language in submitted articles must be clear, correct, and unambiguous. Any typographical or grammatical errors should be corrected at revision, so please note any specific errors here.

Reviewer #1: Yes

Reviewer #2: Yes

Reviewer #3: Yes

Reviewer #4: Yes

**********

5. Review Comments to the Author

Please use the space provided to explain your answers to the questions above. You may also include additional comments for the author, including concerns about dual publication, research ethics, or publication ethics. (Please upload your review as an attachment if it exceeds 20,000 characters)

Reviewer #1: Overall assessment

This manuscript makes a strong and timely contribution to the vaccine delivery costing literature by presenting a detailed microcosting analysis of a nationwide typhoid conjugate vaccine (TCV) campaign in Burkina Faso. Large-scale, empirically grounded costing studies of vaccination campaigns—particularly in low-income and insecure settings—remain relatively rare, and this paper substantially advances the evidence base.

The nationwide scope, use of a bottom-up microcosting approach across administrative levels, and explicit attention to cost variation by delivery strategy and security context are major strengths. The study is well aligned with the article’s stated objectives and is highly relevant for immunization program planners and financing partners. Overall, the manuscript is suitable for publication after moderate revisions focused on clarity, transparency, and interpretation, rather than expansion into cost-effectiveness analysis.

Major strengths

1. Substantive contribution through microcosting

The use of a microcosting approach across health facility, district, regional, and national levels is a major strength and the paper’s core contribution. Few published studies capture delivery costs at this level of granularity for a nationwide campaign of this scale. The approach is appropriate, clearly motivated, and well matched to the study objectives.

2. Scale and empirical rigor

The analysis is grounded in a campaign that delivered over 10 million doses, drawing on data from multiple administrative levels. This scale gives the findings strong empirical credibility and makes the resulting unit costs highly policy-relevant. The inclusion of both financial and economic costs further strengthens the analysis.

3. Policy relevance and operational insight

The disaggregation of costs by:

• delivery strategy (fixed, mobile, temporary),

• geography (urban vs. rural),

• and security context

provides actionable insights for campaign design and budgeting. Identifying human resources and per diem payments as dominant cost drivers is consistent with prior campaign experience and reinforces the practical value of the findings.

4. Clear presentation of unit costs

Reporting costs per dose with confidence intervals and benchmarking financial costs against Gavi operational support ranges enhances interpretability and usability for decision-makers. The results are presented clearly and concisely.

Suggestions for improvement

These comments are intended to strengthen transparency and interpretability, not to change the study’s scope.

1. Clarify key microcosting assumptions

The manuscript would benefit from additional detail on:

• How shared or overhead costs (e.g., planning, supervision) were allocated across doses or administrative levels.

• Methods used to annualize capital costs and assumptions about useful life.

• How staff time and opportunity costs were valued for economic cost estimates.

These clarifications could be addressed succinctly in a methods appendix or supplementary table and would improve reproducibility.

2. Expand description of uncertainty estimation

While confidence intervals are reported, the paper should briefly explain:

• The statistical method used to generate them (e.g., bootstrapping, sampling-based variance).

• What sources of uncertainty are captured (sampling only vs. parameter uncertainty).

This would strengthen readers’ confidence in the precision of the estimates.

3. Improve discussion of generalizability

Given the unique security and logistical context of Burkina Faso, the discussion could more explicitly address:

• Which cost components are likely context-specific.

• Which findings (e.g., dominance of HR and per diems) are likely transferable to other low-income settings.

This would help readers appropriately interpret and apply the results elsewhere.

4. Minor clarity and consistency issues

A careful editorial pass could improve:

• Consistency in terminology across sections (e.g., delivery strategy labels).

• Clearer signposting between financial vs. economic cost results.

• Minor typographical and formatting issues.

Conclusion

This is a high-quality costing study that fills an important gap in the literature on vaccine campaign delivery costs. Its primary contribution lies in the depth and rigor of the microcosting analysis, not in extending to cost-effectiveness modeling—and it should be evaluated on those terms. With targeted revisions to improve methodological transparency and discussion, the manuscript will provide a valuable reference for policymakers, donors, and researchers planning future vaccination campaigns.

Reviewer #2: This manuscript presents a technically sound and well-executed cost analysis of a nationwide typhoid conjugate vaccine campaign in Burkina Faso. The retrospective micro-costing approach, conducted from the government perspective across multiple administrative levels; 50 health facilities, 8 districts, 5 regions, and the national level, is appropriate for the study objectives. The data support the conclusions regarding average financial and economic cost per dose, the identification of human resources and per diem payments as key cost drivers, and observed cost variation by geography, delivery strategy, and security context.

The statistical analyses are appropriate and clearly reported, including weighted cost estimates and 95% confidence intervals, with stratified analyses by urban–rural setting and delivery strategy. Ethical approval and informed consent procedures are documented, and data availability is addressed in line with PLOS ONE requirements. Although, minor clarifications may be recommended to harmonize the Data Availability Statement to briefly describe the method used to derive confidence intervals and clarify the potential impact of replacing inaccessible health facilities due to security constraints. Overall, the manuscript meets PLOS ONE’s criteria for publication, and no substantive scientific concerns remain.

Reviewer #3: This manuscript presents a microcosting analysis of Burkina Faso's national typhoid conjugate vaccine (TCV) campaign conducted in January 2025, which reached 10.5 million children aged 9 months to 14 years. The study estimates financial costs at $0.47 per dose and economic costs at $2.16 per dose, demonstrating that campaign delivery costs fall within Gavi operational support ranges. The multi-level sampling approach and stratification by geography, delivery strategy, and security context provide valuable granularity for policy planning.This addresses an important evidence gap, as TCV costing data from francophone Africa are extremely limited despite high typhoid burden. The methodology is generally sound and findings have clear policy relevance for ministries of health, Gavi, and WHO. However, several methodological clarifications and expansions are needed before publication.

Major comments

1. Inconsistent Dose Denominators

Issue: The manuscript reports conflicting total dose numbers:

Abstract and line 197: 10,552,806 doses

Abstract line 38: "10.5 million doses"

Table 2: 11,422,855 doses nationally

Impact: All per-dose cost calculations depend on the correct denominator. This inconsistency undermines confidence in the primary outcome.

Please Reconcile these figures and explain any discrepancy (e.g., incomplete reporting from non-sampled districts)

Use consistent denominator throughout

Recalculate all per-dose costs if necessary

Verify Table 3 calculations align with corrected denominators

2. Uncertainty Quantification Insufficient

Issue: 95% confidence intervals are presented but methodology is not described. No sensitivity analyses are provided despite multiple assumptions (discount rate, volunteer time valuation, vehicle replacement costs, useful life).

I suggest an additional Methods subsection describing CI calculation (bootstrap? parametric assumptions? accounting for clustering?)

Conduct one-way sensitivity analyses on key parameters:

Discount rate (0%, 3%, 5%)

Volunteer wage assumptions

Vehicle/equipment replacement costs

3. Capital Cost Treatment and Shared Infrastructure

Issue: The authors acknowledge excluding "depreciation of long-term infrastructure" (line 309-310) but do not specify which items. It's unclear whether this represents:

Incremental campaign costs only, or

Full economic costs excluding pre-existing infrastructure

Impact: Affects comparability with other studies and interpretation of economic costs.

Specify which capital items were excluded (cold chain equipment? storage facilities? EPI buildings?)

State clearly whether the perspective is incremental campaign cost vs. average cost including shared infrastructure

Justify exclusions based on whether infrastructure was campaign-specific or shared with routine immunization

If feasible, provide sensitivity estimate including shared infrastructure costs.

MINOR CONCERNS

Methods and Reporting

Line 72: Specify which TCV vaccine (Typbar TCV® or TYPHIBEV®) was used in Burkina Faso

Lines 97-98: Standardize terminology as "microcosting" (no hyphen) throughout

Line 120: Describe replacement protocol for 5 inaccessible CSPS more rigorously. What specific matching criteria were used? Conduct sensitivity analysis excluding these sites.

Line 153: Clarify how oral consent was documented

Add to Methods: "Reporting follows CHEERS 2022 guidelines for economic evaluations" and include completed checklist as supplementary file

Reviewer #4: Title: Cost analysis of a nationwide typhoid conjugate vaccine campaign in Burkina Faso

This study is very important in ensuring that scarce resources are judiciously used especially in resource limited country like Burkina Faso.

This is my comment.

The definition of financial cost and economic cost should be included in the introduction because it will give the reader a better understanding of the cost estimate of administering the vaccine in Burkina Faso.

**********

6. PLOS authors have the option to publish the peer review history of their article (what does this mean?). If published, this will include your full peer review and any attached files.

If you choose “no”, your identity will remain anonymous but your review may still be made public.

**Do you want your identity to be public for this peer review?** For information about this choice, including consent withdrawal, please see our Privacy Policy.

Reviewer #1: No

Reviewer #2: **Yes:** Idowu Michael Ariyibi

Reviewer #3: No

Reviewer #4: **Yes:** Dr Abdulahi Ayanwale

**********

[NOTE: If reviewer comments were submitted as an attachment file, they will be attached to this email and accessible via the submission site. Please log into your account, locate the manuscript record, and check for the action link "View Attachments". If this link does not appear, there are no attachment files.]

To ensure your figures meet our technical requirements, please review our figure guidelines: https://journals.plos.org/plosone/s/figures

You may also use PLOS’s free figure tool, NAAS, to help you prepare publication quality figures: https://journals.plos.org/plosone/s/figures#loc-tools-for-figure-preparation.

NAAS will assess whether your figures meet our technical requirements by comparing each figure against our figure specifications.

Attachment Submitted filename: PLOS Article review on Vaccine Burkina Faso.odt

# Author comment: Author response to Decision Letter 1

**DOI:** 10.1371/journal.pone.0351148.r002

22 Apr 2026

Dear Editor,

We thank you, the PlosOne editorial team and all the reviewers for the insightful and constructive comments to our submission. We have revised the manuscript substantially in response to the comments, to enhance its clarity, transparency, and interpretability. Below, we respond to each comment point-by-point.

Editor and reviewers comments are reproduced in regular font, followed by our responses in bold; important substantive changes to the manuscript are also included in italic.

Editor comments

Thank you for the study; it is quite an interesting manuscript to read. However, I have some few concerns.

First, there is a need to discuss what 'financial and economic cost' means. You need to bring that in your introduction and also state how it was determined in the method section, because you used it quite a lot in the abstract.

Also, there are inconsistencies in the denominator used, so you need to revisit that and make it consistent.

We added the definition of financial cost and economic cost in the introduction section and explain how they were estimated in the method section and in supporting information.

Also, we thank the editor for identifying this inconsistency in the denominator. The correct one is 10,552,806 doses, corresponding to the validated dataset used for all cost analyses.

Review Comments to the Author

Reviewer #1:

We thank the reviewer for the encouraging and thoughtful review, and for recognizing the major strengths, and policy relevance of the study.

1. Clarify key microcosting assumptions

The manuscript would benefit from additional detail on:

• How shared or overhead costs (e.g., planning, supervision) were allocated across doses or administrative levels.

Response to Reviewer #1: The purpose of the study is to estimate TCV campaign specific costs. We estimate these costs at each administrative level by quantifying ressources used for each campaign activity. For shared resources, tracing factors (allocation rules) were used to attribute costs to TCV campaign. These tracing factors included staff time allocated to the campaign, percentage of vehicle and equipment capacity utilized during the campaign, or the number of doses and the volume of other vaccines administered or interventions delivered as part of the campaign.

• Methods used to annualize capital costs and assumptions about useful life.

Response to Reviewer #1: To reflect their use over multiple years, capital items (e.g. cold chain equipment, vehicles, and durable equipment) were annualized using an annualization factor (see formula below) with a discount rate (r) and an assumed useful life (n) for capital items. The annualized cost was allocated to the campaign based on the proportion of time the asset was used during campaign activities.

• How staff time and opportunity costs were valued for economic cost estimates.

Response to the reviewer #1: For economic cost estimates, staff time was valued using an opportunity cost approach. Time allocated to campaign activities was obtained through interviews with health personnel and program managers and multiplied by the corresponding gross salary to estimate the economic value of personnel time.

These clarifications could be addressed succinctly in a methods appendix or supplementary table and would improve reproducibility.

Response to the reviewer #1: We added a supplementary file explaining these clarifications on the estimation of the campaign costs.

2. Expand description of uncertainty estimation

While confidence intervals are reported, the paper should briefly explain:

• The statistical method used to generate them (e.g., bootstrapping, sampling-based variance).

• What sources of uncertainty are captured (sampling only vs. parameter uncertainty).

This would strengthen readers’ confidence in the precision of the estimates.

We have revised the Methods section (lines 158-166) to clearly describe how confidence intervals were generated. Specifically, 95% confidence intervals were calculated using sampling-based variance derived from the study sample, with standard errors estimated from the variability in cost estimates across sampled administrative levels. Confidence intervals were constructed using the normal approximation. These intervals capture sampling uncertainty related to the selection of study sites, but do not incorporate parameter uncertainty. Therefore we conducted one-way sensitivity analyses on key parameters (discount rate, volunteer wage and vehicle and equipment replacement costs). This clarification has been added to the Methods section: «Uncertainty around cost estimates was assessed using sampling-based variance derived from the study sample. Standard errors were calculated based on the variability in cost estimates across the sampled administrative levels. 95% confidence intervals were constructed using the normal approximation. Parameter uncertainty was explored through one-way sensitivity analysis on key assumptions, including the discount rate, valuation of volunteer time, and replacement costs of capital items (supplementary file ). Also, we conduct a scenario analysis by removing the replaced facilities due to security constraints from the analysis.»

3. Improve discussion of generalizability

Given the unique security and logistical context of Burkina Faso, the discussion could more explicitly address:

• Which cost components are likely context-specific.

• Which findings (e.g., dominance of HR and per diems) are likely transferable to other low-income settings.

This would help readers appropriately interpret and apply the results elsewhere.

We appreciated the suggestion and have expanded the Discussion section to include cost components likely context-specific and those transferable to low-and-middle income countries (see lines 332-338): «The generalizability of these findings should be interpreted in light of the specific contextual features of Burkina Faso. While some cost drivers—such as increased transport and logistical costs in conflict-affected areas—are context-specific to Burkina Faso, key findings are likely transferable. In particular, the dominance of human resources and per diem payments as major cost drivers, as well as variations by delivery strategy and economies of scale, are consistent with evidence from other low-income settings.»

4. Minor clarity and consistency issues

A careful editorial pass could improve:

• Consistency in terminology across sections (e.g., delivery strategy labels).

• Clearer signposting between financial vs. economic cost results.

• Minor typographical and formatting issues.

All minor comments have been addressed as follows: We have improved the clarity of presentation by consistently distinguishing between financial and economic costs throughout the manuscript. Separate subheadings and explicit terminology are now used to guide the reader, and additional explanations have been included to clarify the conceptual differences between these two cost measures.

Reviewer #2:

We thank the reviewer for the constructive review of the manuscript. Below we address the clarifications suggested.

Briefly describe the method used to derive confidence intervals:

We have revised the Methods section (lines 158-166) to clearly describe how confidence intervals were generated. Specifically, 95% confidence intervals were calculated using sampling-based variance derived from the study sample, with standard errors estimated from the variability in cost estimates across sampled administrative levels. Confidence intervals were constructed using the normal approximation. These intervals capture sampling uncertainty related to the selection of study sites, but do not incorporate parameter uncertainty. Therefore we conducted one-way sensitivity analyses on key parameters (discount rate, volunteer wage and vehicle and equipment replacement costs). This clarification has been added to the Methods section: «Uncertainty around cost estimates was assessed using sampling-based variance derived from the study sample. Standard errors were calculated based on the variability in cost estimates across the sampled administrative levels. 95% confidence intervals were constructed using the normal approximation. Parameter uncertainty was explored through one-way sensitivity analysis on key assumptions, including the discount rate, valuation of volunteer time, and replacement costs of capital items (supplementary file ). Also, we conduct a scenario analysis by removing the replaced facilities due to security constraints from the analysis.»

Clarify the potential impact of replacing inaccessible health facilities due to security constraints.

In the study limitations section (lines 368-373) we have a short paragraph acknowledging that five health facilities were inaccessible due to security constraints and were replaced with accessible facilities considered comparable in terms of cost structure or campaign implementation (similar catchment area population, number of doses delivered). Moreover, we added a scenario analysis excluding these health facilities. This analysis indicated that their replacement had no substantial impact on either financial or economic costs estimates (See lines 270-274).

Reviewer #3:

We thank the reviewer for the positive evaluation of the manuscript’s relevance, strengths, as well as for the detailed and constructive suggestions.

Below we address the suggested comments.

1. Inconsistent Dose Denominators

Issue: The manuscript reports conflicting total dose numbers:

Abstract and line 197: 10,552,806 doses

Abstract line 38: "10.5 million doses"

Table 2: 11,422,855 doses nationally

Impact: All per-dose cost calculations depend on the correct denominator. This inconsistency undermines confidence in the primary outcome.

Please Reconcile these figures and explain any discrepancy (e.g., incomplete reporting from non-sampled districts)

Use consistent denominator throughout

Recalculate all per-dose costs if necessary

Verify Table 3 calculations align with corrected denominators

We thank the reviewer for identifying this inconsistency. The correct denominator is 10,552,806 doses, corresponding to the validated dataset used for all cost analyses. The higher figure previously reported in Table 2 (11,422,855 doses) reflected an earlier, uncleaned version of the national dataset and has now been corrected. All per-dose cost estimates, including those in the Abstract and Table 3, were calculated using the correct denominator; therefore, no recalculation was required.

2. Uncertainty Quantification Insufficient

Issue: 95% confidence intervals are presented but methodology is not described. No sensitivity analyses are provided despite multiple assumptions (discount rate, volunteer time valuation, vehicle replacement costs, useful life).

I suggest an additional Methods subsection describing CI calculation (bootstrap? parametric assumptions? accounting for clustering?)

Conduct one-way sensitivity analyses on key parameters:

Discount rate (0%, 3%, 5%)

Volunteer wage assumptions

Vehicle/equipment replacement costs

We have revised the Methods section to clearly describe how confidence intervals were generated. Specifically, 95% confidence intervals were calculated using sampling-based variance derived from the study sample, with standard errors estimated from the variability in cost estimates across sampled administrative levels. Confidence intervals were constructed using the normal approximation. These intervals capture sampling uncertainty related to the selection of study sites, but do not incorporate parameter uncertainty. Therefore we conducted one-way sensitivity analyses on key parameters (discount rate, volunteer wage and vehicle and equipment replacement costs). This clarification has been added to the Methods section: «Uncertainty around cost estimates was assessed using sampling-based variance derived from the study sample. Standard errors were calculated based on the variability in cost estimates across the sampled administrative levels. 95% confidence intervals were constructed using the normal approximation. Parameter uncertainty was explored through one-way sensitivity analysis on key assumptions, including the discount rate, valuation of volunteer time, and replacement costs of capital items (supplementary file ). Also, we conduct a scenario analysis by removing the replaced facilities due to security constraints from the analysis.»

3. Capital Cost Treatment and Shared Infrastructure

Issue: The authors acknowledge excluding "depreciation of long-term infrastructure" (line 309-310) but do not specify which items. It's unclear whether this represents:

Incremental campaign costs only, or

Full economic costs excluding pre-existing infrastructure

Impact: Affects comparability with other studies and interpretation of economic costs.

Specify which capital items were excluded (cold chain equipment? storage facilities? EPI buildings?)

State clearly whether the perspective is incremental campaign cost vs. average cost including shared infrastructure

Justify exclusions based on whether infrastructure was campaign-specific or shared with routine immunization

If feasible, provide sensitivity estimate including shared infrastructure costs.

We have revised the manuscript to clarify the costing perspective and the treatment of capital and shared infrastructure costs (lines 333-334). The analysis adopts an incremental campaign costing perspective, aiming to capture the additional resources required to implement the TCV campaign, rather than the full economic cost of the immunization system.

Accordingly, long-term infrastructure costs were excluded, specifically EPI buildings and office infrastructure. These items were excluded because they were not procured nor expanded specifically for the campaign and are shared with routine immunization activities. We have now explicitly stated this in the Methods section and clarified that the reported economic costs include annualized capital costs for campaign-specific items only (e.g., vehicles and equipment mobilized for the campaign where applicable).

MINOR CONCERNS

Methods and Reporting

Line 72: Specify which TCV vaccine (Typbar TCV® or TYPHIBEV®) was used in Burkina Faso

In Burkina Faso, TYPHIBEV® was used for the campaign.

Lines 97-98: Standardize terminology as "microcosting" (no hyphen) throughout

We used a standard terminology «microcosting» throughout the manuscript.

Line 120: Describe replacement protocol for 5 inaccessible CSPS more rigorously. What specific matching criteria were used? Conduct sensitivity analysis excluding these sites.

We added a sentence in the methods section to describe more rigorously the replacement protocol of inaccessible facilities as follows (lines 123-127): «Due to security concerns, we replaced five inaccessible CSPS with alternative facilities with similar characteristics. The matching criteria were based on sampling weights derived from catchment area population size and the number of doses delivered, as well as stratification by urban versus rural location.»

Furthermore we conducted a scenario analysis excluding these sites This analysis indicated that there was no substantial impact on either financial or economic costs estimates (See lines 270-274).

Line 153: Clarify how oral consent was documented

Add to Methods: "Reporting follows CHEERS 2022 guidelines for economic evaluations" and include completed checklist as supplementary file

Oral consent was obtained prior to each interview after the study objectives and procedures were read aloud to participants. Consent was documented by the data collector’s signature on a standardized form confirming that the information had been provided and that the participant agreed to take part in the study. Also, we added to methods section, a sentence related to CHEERS guidelines and included completed CHEERS checklist as a supplementary file.

Reviewer #4:

We thank the reviewer for the constructive review of the manuscript

The definition of financial cost and economic cost should be included in the introduction because it will give the reader a better understanding of t

Attachment Submitted filename: Responses to reviewers.docx

# Peer review: Decision Letter 1

**DOI:** 10.1371/journal.pone.0351148.r003

**Authors:** Leonard Uzairue

10 May 2026

PONE-D-25-66817R1

Cost analysis of a nationwide typhoid conjugate vaccine campaign in Burkina Faso

PLOS One

Dear Dr. Koulidiati,

Thank you for submitting your manuscript to PLOS ONE. After careful consideration, we feel that it has merit but does not fully meet PLOS ONE’s publication criteria as it currently stands. Therefore, we invite you to submit a revised version of the manuscript that addresses the points raised during the review process.

Please submit your revised manuscript by Jun 24 2026 11:59PM. If you will need more time than this to complete your revisions, please reply to this message or contact the journal office at plosone@plos.org. When you're ready to submit your revision, log on to https://www.editorialmanager.com/pone/ and select the 'Submissions Needing Revision' folder to locate your manuscript file.

Please include the following items when submitting your revised manuscript:

- A letter that responds to each point raised by the academic editor and reviewer(s). You should upload this letter as a separate file labeled 'Response to Reviewers'.
- A marked-up copy of your manuscript that highlights changes made to the original version. You should upload this as a separate file labeled 'Revised Manuscript with Track Changes'.
- An unmarked version of your revised paper without tracked changes. You should upload this as a separate file labeled 'Manuscript'.

If you would like to make changes to your financial disclosure, please include your updated statement in your cover letter. Guidelines for resubmitting your figure files are available below the reviewer comments at the end of this letter.

If applicable, we recommend that you deposit your laboratory protocols in protocols.io to enhance the reproducibility of your results. Protocols.io assigns your protocol its own identifier (DOI) so that it can be cited independently in the future. For instructions see: https://journals.plos.org/plosone/s/submission-guidelines#loc-laboratory-protocols. Additionally, PLOS ONE offers an option for publishing peer-reviewed Lab Protocol articles, which describe protocols hosted on protocols.io. Read more information on sharing protocols at https://plos.org/protocols?utm_medium=editorial-email&utm_source=authorletters&utm_campaign=protocols.

As the corresponding author, your ORCID iD is verified in the submission system and will appear in the published article. PLOS supports the use of ORCID, and we encourage all coauthors to register for an ORCID iD and use it as well. Please encourage your coauthors to verify their ORCID iD within the submission system before final acceptance, as unverified ORCID iDs will not appear in the published article. *Only* the individual author can complete the verification step; PLOS staff *cannot* verify ORCID iDs on behalf of authors.

We look forward to receiving your revised manuscript.

Kind regards,

Leonard Ighodalo Uzairue, PhD

Academic Editor

PLOS One

Journal Requirements:

1. If the reviewer comments include a recommendation to cite specific previously published works, please review and evaluate these publications to determine whether they are relevant and should be cited. There is no requirement to cite these works unless the editor has indicated otherwise.

2. Please review your reference list to ensure that it is complete and correct. If you have cited papers that have been retracted, please include the rationale for doing so in the manuscript text, or remove these references and replace them with relevant current references. Any changes to the reference list should be mentioned in the rebuttal letter that accompanies your revised manuscript. If you need to cite a retracted article, indicate the article’s retracted status in the References list and also include a citation and full reference for the retraction notice.

[Note: HTML markup is below. Please do not edit.]

Reviewers' comments:

Reviewer's Responses to Questions

**Comments to the Author**

1. If the authors have adequately addressed your comments raised in a previous round of review and you feel that this manuscript is now acceptable for publication, you may indicate that here to bypass the “Comments to the Author” section, enter your conflict of interest statement in the “Confidential to Editor” section, and submit your "Accept" recommendation.

Reviewer #3: All comments have been addressed

Reviewer #4: All comments have been addressed

**********

2. Is the manuscript technically sound, and do the data support the conclusions?

The manuscript must describe a technically sound piece of scientific research with data that supports the conclusions. Experiments must have been conducted rigorously, with appropriate controls, replication, and sample sizes. The conclusions must be drawn appropriately based on the data presented.

Reviewer #3: Yes

Reviewer #4: Yes

**********

3. Has the statistical analysis been performed appropriately and rigorously?

Reviewer #3: Yes

Reviewer #4: Yes

**********

4. Have the authors made all data underlying the findings in their manuscript fully available?

The PLOS Data policy requires authors to make all data underlying the findings described in their manuscript fully available without restriction, with rare exception (please refer to the Data Availability Statement in the manuscript PDF file). The data should be provided as part of the manuscript or its supporting information, or deposited to a public repository. For example, in addition to summary statistics, the data points behind means, medians and variance measures should be available. If there are restrictions on publicly sharing data—e.g. participant privacy or use of data from a third party—those must be specified.

Reviewer #3: Yes

Reviewer #4: Yes

**********

5. Is the manuscript presented in an intelligible fashion and written in standard English?

PLOS ONE does not copyedit accepted manuscripts, so the language in submitted articles must be clear, correct, and unambiguous. Any typographical or grammatical errors should be corrected at revision, so please note any specific errors here.

Reviewer #3: Yes

Reviewer #4: Yes

**********

6. Review Comments to the Author

Please use the space provided to explain your answers to the questions above. You may also include additional comments for the author, including concerns about dual publication, research ethics, or publication ethics. (Please upload your review as an attachment if it exceeds 20,000 characters)

Reviewer #3: The authors have substantively improved the manuscript and addressed most prior concerns. Remaining issues are mostly editorial

Reviewer #4: The topic Cost analysis of a nationwide typhoid conjugate vaccine campaign in Burkina Faso.

The issues of definition of some terms were addressed and the define them in the introduction section of the paper.

I believe that the article can be published.

**********

7. PLOS authors have the option to publish the peer review history of their article (what does this mean?). If published, this will include your full peer review and any attached files.

If you choose “no”, your identity will remain anonymous but your review may still be made public.

**Do you want your identity to be public for this peer review?** For information about this choice, including consent withdrawal, please see our Privacy Policy.

Reviewer #3: No

Reviewer #4: No

**********

[NOTE: If reviewer comments were submitted as an attachment file, they will be attached to this email and accessible via the submission site. Please log into your account, locate the manuscript record, and check for the action link "View Attachments". If this link does not appear, there are no attachment files.]

To ensure your figures meet our technical requirements, please review our figure guidelines: https://journals.plos.org/plosone/s/figures

You may also use PLOS’s free figure tool, NAAS, to help you prepare publication quality figures: https://journals.plos.org/plosone/s/figures#loc-tools-for-figure-preparation.

NAAS will assess whether your figures meet our technical requirements by comparing each figure against our figure specifications.

Attachment Submitted filename: PLOS reviewer.docx

# Author comment: Author response to Decision Letter 2

**DOI:** 10.1371/journal.pone.0351148.r004

12 May 2026

Dear Editor,

We thank you, the PlosOne editorial team and all the reviewers for the constructive comments to our submission. Below, we respond to each comment.

Editor and reviewers comments are reproduced in regular font, followed by our responses in bold.

Review Comments to the Author

Reviewer #3: The authors have substantively improved the manuscript and addressed most prior concerns. Remaining issues are mostly editorial

We thank the reviewer for their positive assessment and constructive comments. We have carefully reviewed the manuscript and addressed all remaining editorial issues, including formatting consistency, tense agreement, and citation style. Changes are highlighted in the tracked-changes version of the manuscript.

Reviewer #4: The topic Cost analysis of a nationwide typhoid conjugate vaccine campaign in Burkina Faso.

The issues of definition of some terms were addressed and the define them in the introduction section of the paper.

I believe that the article can be published.

We thank the reviewer for their review and positive recommendation.

Attachment Submitted filename: Responses to reviewers_V2.docx

# Editor report: Decision Letter 2

**DOI:** 10.1371/journal.pone.0351148.r005

**Authors:** Leonard Uzairue

25 May 2026

Cost analysis of a nationwide typhoid conjugate vaccine campaign in Burkina Faso

PONE-D-25-66817R2

Dear Koulidiati,

We’re pleased to inform you that your manuscript has been judged scientifically suitable for publication and will be formally accepted for publication once it meets all outstanding technical requirements.

Within one week, you’ll receive an e-mail detailing the required amendments. When these have been addressed, you’ll receive a formal acceptance letter and your manuscript will be scheduled for publication.

An invoice will be generated when your article is formally accepted. Please note, if your institution has a publishing partnership with PLOS and your article meets the relevant criteria, all or part of your publication costs will be covered. Please make sure your user information is up-to-date by logging into Editorial Manager at Editorial Manager® and clicking the ‘Update My Information' link at the top of the page. For questions related to billing, please contact billing support.

If your institution or institutions have a press office, please notify them about your upcoming paper to help maximize its impact. If they’ll be preparing press materials, please inform our press team as soon as possible -- no later than 48 hours after receiving the formal acceptance. Your manuscript will remain under strict press embargo until 2 pm Eastern Time on the date of publication. For more information, please contact onepress@plos.org.

Kind regards,

Leonard Ighodalo Uzairue, PhD

Academic Editor

PLOS One

Additional Editor Comments (optional):

Reviewers' comments:

# Editor report: Acceptance letter

**DOI:** 10.1371/journal.pone.0351148.r006

**Authors:** Leonard Uzairue

PONE-D-25-66817R2

PLOS One

Dear Dr. Koulidiati,

I'm pleased to inform you that your manuscript has been deemed suitable for publication in PLOS One. Congratulations! Your manuscript is now being handed over to our production team.

At this stage, our production department will prepare your paper for publication. This includes ensuring the following:

* All references, tables, and figures are properly cited

* All relevant supporting information is included in the manuscript submission,

* There are no issues that prevent the paper from being properly typeset

You will receive further instructions from the production team, including instructions on how to review your proof when it is ready. Please keep in mind that we are working through a large volume of accepted articles, so please give us a few days to review your paper and let you know the next and final steps.

Lastly, if your institution or institutions have a press office, please let them know about your upcoming paper now to help maximize its impact. If they'll be preparing press materials, please inform our press team within the next 48 hours. Your manuscript will remain under strict press embargo until 2 pm Eastern Time on the date of publication. For more information, please contact onepress@plos.org.

You will receive an invoice from PLOS for your publication fee after your manuscript has reached the completed accept phase. If you receive an email requesting payment before acceptance or for any other service, this may be a phishing scheme. Learn how to identify phishing emails and protect your accounts at https://explore.plos.org/phishing.

If we can help with anything else, please email us at customercare@plos.org.

Thank you for submitting your work to PLOS ONE and supporting open access.

Kind regards,

PLOS ONE Editorial Office Staff

on behalf of

Dr. Leonard Ighodalo Uzairue

Academic Editor

PLOS One
